# Socioeconomic Disparity in Later-Year Group Trajectories of Depressive Symptoms: Role of Health and Social Engagement Change

**DOI:** 10.3390/ijerph14060588

**Published:** 2017-06-01

**Authors:** Hyunjoo Lee, Sojung Park, Eunsun Kwon, Joonyoung Cho

**Affiliations:** 1 Department of Social Welfare, Daegu University, 201 Deagudae-ro, Gyeongsangbuk-do, Gyeongsan-si 38453, Korea; 2 George Warren Brown School of Social Work at Washington University in Saint Louis, One Brookings Drive, Saint Louis, MO 63130, USA; spark30@wustl.edu (S.P.); joonyoungcho@wustl.edu (J.C.); 3 Center for Social Science, Seoul National University, 1 Gwanak-ro, Gwanak-gu, Seoul 08826, Korea; ek2218@columbia.edu

**Keywords:** later-year depressive symptoms, group trajectories, poverty status, health and social engagement

## Abstract

This study explored heterogeneous change patterns of South Korean older adults’ depressive symptoms by poverty status, focusing on health status and social engagement changes. We used data from four waves (2006–2012) of the Korean Longitudinal Study of Aging (KLoSA). Our sample contained 2461 poor and 1668 non-poor individuals. All were 65 years old or older at baseline. We used latent class growth analysis to identify trajectory groups’ depressive symptoms. Multinomial logistic regression was used to examine how a range of changes in health conditions and social engagement was associated with trajectories among poor and non-poor participants. Among the poor, five heterogeneous trajectories with clear patterns were identified: high-to-moderate, stable-high, slightly-increasing, steeply-increasing, and stable-low. Among non-poor, high-to-moderate, steeply-increasing, and stable-low groups were found. A decrease in health conditions was the most vulnerable subgroup’s (steeply-increasing) primary risk factor. Poor older adults who reduced participation in, or decreased contact with, social networks were likely to belong to the steeply-increasing group. Our study provides impetus for organizational and/or environmental support systems to facilitate social engagement among poor older adults. Future research should examine whether the significance of social engagement among poor elders applies in less-developed and developed countries.

## 1. Introduction

The influence of socio-economic disparity in later-year depressive symptoms has been well-established in both developing and industrialized countries [1]. Guided by a life course perspective, growing longitudinal research has begun to identify vulnerable subgroups whose trajectory of depressive symptoms may diverge from the average. This emphasis on identifying subgroups has important clinical and policy implications for targeted interventions.

In this new line of research, the inverse relationship between poverty and depressive symptoms is consistent: poor older adults show consistently higher levels of or increases in depressive symptoms. Existing evidence suggests older adults exhibit heterogeneous patterns of long-term change, partly because socioeconomic position (SEP) has a direct causal relationship with future status [2,3] that varies by severity and stability [4]. Studies typically identify four subgroups: stable low, persistently high, and dynamic change groups (increasing and decreasing) [5,6,7], but some have found as many as six [8].

The seemingly persistent effect of low income or poverty is an important concern, although not all poor older adults show consistent or increasing levels of depressive symptoms; some exhibit improving patterns or maintain low levels of depressive symptoms. To date, little is known about possible heterogeneous change patterns among poor older adults or how their change patterns may differ from non-poor peers. In this study, we examine change patterns of later-year depressive symptoms by poverty status.

South Korea (hereafter Korea) provides a unique context in which to examine poverty-specific trajectories of depressive symptoms and their heterogeneity. Previous studies have found that while disparate patterns of depressive symptom trajectories exist in developed countries, such as the United States and France [4], elderly poverty is not as acute an issue as it is in Korea—a developed country characterized by the world’s highest elderly poverty rate and fastest-aging population [9]. 49.6% of older Korean adults live in poverty—half the median household income of the total population—the highest relative poverty rate among Organization for Economic Cooperation and Development (OECD) countries [10]. While late-life major depression is less prevalent in Asian countries, like China and Japan, it is more prevalent in Korea than in most Western and Asian countries [11] and has become a serious social problem as the country’s pace of socioeconomic and demographic change has outstripped its ability to address elder poverty and related social problems.

The positive relationship between low income and depressive symptoms has long been reported in cross-sectional [12] and longitudinal studies [13,14]. To date, no study has examined the extent of the heterogeneity of depressive symptoms among the poor. Given its unique context—high elderly poverty, rapidly aging population, and limited supportive infrastructure—developing empirical knowledge of longitudinal patterns of depressive symptoms in Korea will provide important research and policy foundations for scholars and practitioners.

In this study, we focused on two predictors of later-year depressive symptoms: health conditions and social engagement. A strong and consistent association between health conditions and later-year depressive symptoms is well-established. Predictors of higher symptom burdens include poor self-rated health [7,8,15], chronic diseases [5,6,8,15], functional impairment [5,6,7,16], and cognitive function [7,16,17]. Indeed, most older adults experience a constellation of co-related, chronic, functional, and mental impairments [18]. However, extant studies have focused on limited aspects of these multiple health conditions and have failed to consider, comprehensively, a range of health factors.

Social engagement refers to the maintenance of social connections and productive participation in social activities [19]. A large body of literature grounded in activity theory has demonstrated that less social participation and interaction are associated with higher levels of depressive symptoms over time [20,21,22,23]. Research based on life-course or life-span theories [24,25] has highlighted the importance of close family relationships and friendships for meeting individuals’ emotional and instrumental support needs and contributing to positive well-being. Generally, studies have found that participation in activities (religious activities, clubs, and political groups, among others) and volunteering are associated with better mental health and reduced levels of depressive symptoms [26]. No studies have examined health and social engagement factors in identifying group trajectories of depressive symptoms. In most studies, these factors are examined as static conditions at baseline; their use in investigating the extent to which changes affect depressive symptom patterns over time has been limited.

This study has two objectives. First, we attempt to identify distinctive trajectories of later-year depressive symptoms over a six-year observation period. We expect to find several subgroups with stable and unstable change patterns. Second, we examine the extent to which changes in health conditions and social engagement factors affect group trajectories of depressive symptoms. We expect deteriorating health conditions and reduced social engagement to be associated with subgroups demonstrating exacerbated depressive symptoms.

To examine these questions, we explored separate models for poor and non-poor at baseline. Since no previous study has examined poverty-based group trajectories of depressive symptoms, we established no specific hypothesis. Based on socioeconomic inequality literature, however, we expect poverty to be associated with worse health conditions [27,28], such that the subgroups with high depressive symptoms will have a higher proportion of respondents who identify as poor than subgroups with lower depressive symptoms.

## 2. Materials and Methods

### 2.1. Data and Sample

We used data from four waves (2006–2012) of the Korean Longitudinal Study of Aging (KLoSA), a nationally-representative bi-annual study of older Korean adults. In the first wave, 10,254 (retention rate = 86.6%); community-dwelling respondents participated in the survey; 8688 in the second; 7920 (retention rate = 81.7%) in the third; and 7486 (retention rate = 80.1%) in the fourth wave. We selected the 4162 respondents aged 65 and older at baseline.

To focus on low-income individuals, we calculated the income-to-needs ratio (INR), dividing disposable income by the poverty thresholds established by the Korea Ministry of Health and Welfare. The poverty thresholds are intended to represent the minimum cost of living and are estimated from data on household income and expenditure measures collected by the Korean National Bureau of Statistics (KOSTAT). The Korea Ministry of Health and Welfare provides detailed annual poverty thresholds varied by family size, age of each household member, and changes in the consumer price index.

We divided the sample into two groups: poor with INR of 1 or lower, and non-poor, excluding those who provided no information on depressive symptoms (*n* = 33). Our final sample included 2461 individuals in the poor group and 1668 individuals in the non-poor group.

### 2.2. Measures

Depressive symptoms: We measured depressive symptoms using the Center for Epidemiological Studies Depression 10-item scale (CES-D 10) [29]. Respondents were asked how often during the last week they had experienced 10 items for affective, somatic, and interpersonal symptoms of depression. These were coded on a four-point scale, from 0 (rarely/none of the time) to 3 (most/all of the time) (α = 0.843 in wave 1, 0.868 in wave 2, 0.875 in wave 3, and 0.870 in wave 4). Total scores ranged from 0 to 30, with higher scores representing more depressive symptoms.

Health status: Health status was assessed for three categories: chronic physical disease, functional disability, and cognitive function. For all health conditions, we created dummy variables for change types. Chronic disease was measured by number of doctor diagnoses. Three dummy groups were created: constantly not having disease (0); constantly having disease (1); and status changed (2). Functional disability was assessed by the sum of difficulties with seven Activities of Daily Living (ADL) skills and 10 Instrumental Activities of Daily Living (IADL) skills. Dummy variables of functional disability change were: not having disability (0); constantly having disability (1); increased disability (2); and decreased disability (3). To measure cognitive function, we used the Korean version of the Mini-Mental State Examination (K-MMSE) (0–30). A score of less than 17 indicates suspected dementia; 18–23 low cognitive function; and 24 more normal cognitive function [30]. We created the following dummy variables for cognitive functional change: constantly normal (0); constantly low or suspected dementia (1); improved cognitive function (2); and declined cognitive function (3).

Social engagement: Social engagement was measured with two indicators: social participation and contact with a close social network. Social participation was assessed by asking whether the respondent participated in seven activities: religious services, social clubs at a senior center, regular sports or cultural clubs, alumni events, village/clan events, volunteer activities, and political or civic organizations. Following previous studies [31], participation in each activity was totaled for a summary score ranging from 0 to 7. We created dummy variables for social participation change: constantly participated (0); constantly no participation (1); increased participation (2); and decreased participation (3).

Contact with social network was assessed as the frequency of contact on a scale of 0–4 (rarely, a few times a year, a few times a month, a few times a week, everyday) with close friends, neighbors, or relatives. Dummy variables for contact frequency change were created: no change in contact frequency (0); increased contact frequency (1); and decreased contact frequency (3).

Covariates: Demographic variables included gender (male, 0; female 1); education was a binary indicator (more than six years of education (0) or lower (1)); using INR, we categorized respondents into four groups based on the length of income status: constantly non-poor or not (0/1); constantly poor or not (0/1); exit from poverty or not (0/1) if poverty status changed from poor to non-poor over the course of the study (0/1); and entry into poverty (0/1) if poverty status changed from non-poor to poor; and marital status (constantly married, 0; constantly not married, 1; marital status changed, 2). Depressive symptoms and log-transformed income values at baseline were included to control for possible reverse causality.

### 2.3. Analysis

To identify heterogeneous subgroups of depressive symptom change, we used longitudinal person-centered analysis. The growth mixture model (GMM) enabled us to identify information about inter-individual differences in intra-individual change involving unobserved heterogeneity within a larger population [32]. As a special case of GMM, we used latent class growth analysis (LCGA) to determine the number of latent classes, including the Lo, Mendell, Rubin Likelihood Ratio Test (LMR-LRT) for statistical testing. For relative comparison, we used model fit indices, such as the Bayesian Information Criterion (BIC) value, sample size-adjusted BIC (SSABIC), and entropy. Smaller BIC and higher entropy values suggest better-fitting models [33]. All parameter estimates for models were obtained using full-information maximum likelihood estimation (FIML). This includes subjects with missing data in the estimate by breaking the likelihood function into components based on missing data patterns [34]. We conducted analyses using Mplus version 5.0 for LCGA.

Data analyses followed three steps. First, descriptive statistical analyses were conducted. Second, we used LCGA to identify distinctive clusters of individual depression trajectories. Given well-established evidence of gender-patterned later-year depressive symptoms [6,17], a conditional LCGA was conducted in which gender was entered as a factor to have a direct effect on the growth factors. Third, multinomial logistic regression modeling was used to explore health status and social engagement as predictors of latent trajectory groups in depressive symptom change. The multinomial logistic regression analysis was based on the assumption that there was no relation between independent variables and unobserved time-constant factors.

## 3. Results

### 3.1. Group Trajectories of Depressive Symptoms by Poverty Status

**Figure 1:**
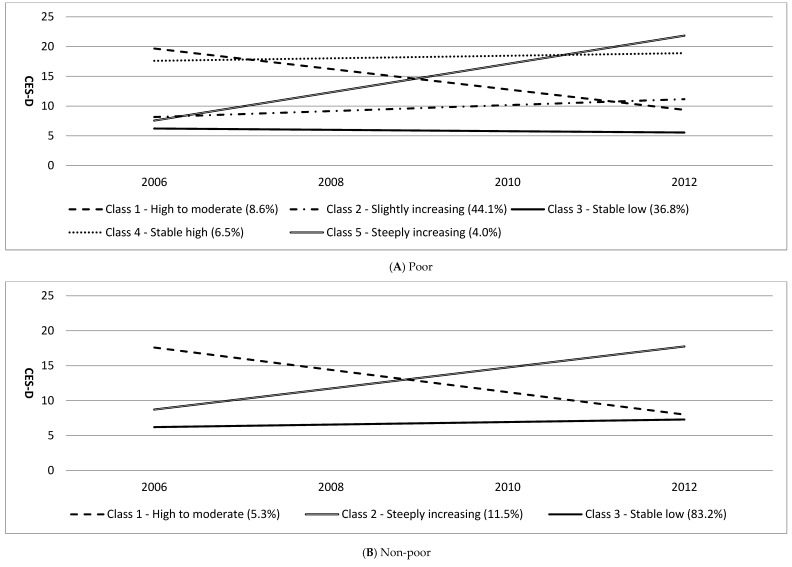
Depressive symptoms group trajectories in older adults (2006–2012).

Based on the five criteria indices and better interpretation of trajectories, we selected the five-group model as the best (Table 1): high-to-moderate (Class 1; *n* = 212, 8.61%), slightly-increasing (Class 2; *n* = 1086, 44.13%), stable-low (Class 3; *n* = 741, 36.77%), stable-high (Class 4; *n* = 160, 6.50%), and steeply-increasing (Class 5; *n* = 98, 3.98%) (Figure 1). High-to-moderate participants exhibited declining patterns of depressive symptoms over time, and stable-high maintained their high level of depressive symptoms. Slightly-increasing and steeply-increasing participants had similar levels of depressive symptoms at baseline; however, depressive symptoms among steeply-increasing participants increased to the highest level among all groups, while slightly-increasing participants demonstrated little change. We identified three groups of non-poor participants: high-to-moderate (Class 1; *n* = 88, 5.3%), steeply-increasing (Class 2; *n* = 192, 11.5%), and stable-low (Class 3; *n* = 1388, 83.2%).

### 3.2. Characteristics of Group Trajectories

Table 1 and Table 2 present characteristics of the depressive symptoms trajectory groups. The stable-high group had the highest proportion of chronic diseases (90.6%) and functional limitations (13.6%) throughout the observed period, and low cognitive function or high dementia risk (79.5%). About a fourth (26.1%) of those in the stable-low group had no chronic conditions, almost three-quarters (72.3%) had no functional diseases, and few had cognitive impairment (13.1%). The steeply-increasing group had the highest proportion of changes in chronic disease (19.4%), increases in functional disability (65.3%), and the lowest proportion experiencing improvement in cognitive function (6.1%). Regarding social engagement, older adults in the steeply increasing group had the highest proportion of decrease in social participation (43.9%) and contact with close social network (42.9%), while the stable-high group had the highest proportion of non-social participation (38.6%). Among the non-poor, the steeply-increasing group had the largest increase in chronic conditions (15.1%) and functional limitations (42.7%); a sharp decrease in cognitive function (26.6%); and the highest proportion of decreased social participation (26.6%) and decreased contact with close social networks (33.9%).

### 3.3. Health and Social Factors of Trajectory Group Membership

We examined the extent to which change patterns in health status and social engagement were associated with depressive symptom trajectories (Table 3). In both poor and non-poor groups, the stable-low subgroup was the reference. Among the poor, those who constantly had chronic conditions, experienced a decrease in functional health, and had constantly low cognitive function and high dementia risk were more likely to fit the stable-high profile. Increased chronic conditions and functional limitations, together with decreased cognitive function and constantly low cognitive function and high dementia risk were associated with the steeply-increasing profile. Improved cognitive function was significantly associated with the high-to-moderate profile (Relative Risk Ratio, RRR = 3.90, *p* < 0.05). Regarding social factors, constant non-participation in, and decreased participation and contact with, social networks were associated with the steeply-increasing group. In sum, results from bi- and multivariate analyses indicate that, among the poor, the two high-risk subgroups were stable-high and steeply-increasing. Risk factors for stable-high included constant chronic conditions and low cognitive function; and for steeply increasing, constantly having health problems, deteriorating health conditions, constant low cognitive function, and reduced social contact and participation.

We carefully examined the diverging trajectory patterns of the two risk groups, stable-high and steeply-increasing, conducting additional analyses using a different reference group (results not shown). When compared with the high-to-moderate group, those with constant chronic disease were more likely to belong in the stable-high group (RRR = 3.207, *p* < 0.05). Moreover, when compared with the slightly-increasing group, constantly having functional disability (RRR = 3.679, *p* < 0.01) and increased disability (RRR = 8.067, *p* < 0.001) were significant predictors of membership in the steeply-increasing group. Constant non-participation in social activities (RRR = 2.356, *p* < 0.05) and reduced participation in social activities (RRR = 3.977, *p* < 0.001) were positively associated with being in the steeply-increasing group.

Among the non–poor (Table 4), constantly having chronic conditions (RRR = 2.425, *p* < 0.01), increased functional limitations (RRR = 3.080, *p* < 0.01), and constantly low cognitive function/dementia risk (RRR = 3.289, *p* < 0.01), together with declines in cognitive function (RRR = 3.697, *p* < 0.01) were significant factors associated with the steeply-increasing group. Regarding social engagement, only decreased contact with social networks was found to be a risk factor for this subgroup.

## 4. Discussion

This study explored heterogeneous change patterns of depressive symptoms among Korean older adults by poverty status, with a focus on changes in health status and social engagement. To the best of our knowledge, this is the first study to examine group trajectories of later-year depressive symptoms by poverty status. Our study contributes three primary findings to the literature on subgroup trajectories of later-year depressive symptoms: (1) depressive symptoms evolve differentially among poor and non-poor older adults; (2) decreasing health conditions are risk factors for both poor and non-poor elderly; and (3) social engagement is an important factor in the development of depressive symptoms trajectories among the poor and non-poor. Given the global public health challenge of later-year depression [1], our findings have implications for both research and policy.

### 4.1. Group Trajectories of Depressive Symptoms by Poverty Status

Our first research question looked at whether different patterns of later-year depressive symptoms would emerge in poor and non-poor groups. Consistent with existing studies, we found stability and change that were divergent and poverty-based.

Underlying these common patterns are important insights: poor older adults show more dynamic patterns of change. The stable-low group was more prevalent among the non-poor (83%) than the poor (37%). As expected, subgroups with persistently high depressive symptoms were identified only among the poor, confirming their relative vulnerability; however, the more dynamic trajectories observed in this group challenge accepted understandings about poverty’s effects on later-year depressive symptoms.

These trends suggest that poor older adults are not necessarily on a uniformly low or worsening, or both, trajectory of depressive symptoms; rather, certain subgroups diverge over time. The experiences of the stable-high and slightly-increasing subgroups drive up the overall level of depressive symptoms. This important empirical knowledge can be used to identify the most vulnerable subgroups among the poor.

### 4.2. Health, Social Engagement, and Symptom Trajectories: Poor vs. Non-Poor

Our second research question asked to what extent health conditions and social engagement factors were associated with depressive symptom trajectories. In this discussion, we focus on the two most vulnerable subgroups, stable-high and steeply-increasing. Among the poor, compared to the stable-low group those in the stable-high group were vulnerable to multiple health conditions with persistent patterns of low health (constant chronic conditions, increase in functional limitations, constantly low cognitive function, and dementia risk). Regarding the steeply-increasing group, worsening health problems (increase in chronic conditions and functional limitations, and cognitive decline) were significant; among the non-poor, this association held. Taken together, these results corroborate our earlier findings: not only is the steeply-increasing group present in both poverty groups, but a decrease in health conditions over time is a primary risk factor regardless of poverty.

The effects of social engagement factors on the poor were pronounced in the most vulnerable trajectory groups, particularly steeply-increasing. Poor older adults who either did not participate in social networks or whose participation had decreased over time were more likely to be in the steeply-increasing group; in the non-poor, similar patterns held, except that the effect of social engagement seemed to be narrower, with only decreased social network contact significant. This finding provides important insight into research and practice. For example, older adults with limited resources are not as prone to engage in meaningful social activities (e.g., volunteering), perhaps because they have fewer opportunities [35]. Social engagement has been discussed as a public health strategy for aging societies [26], a way to achieve successful and productive individual aging while contributing to community and society [36]. However, the push toward active engagement can also be cause for concern: if low-income elders in vulnerable trajectory subgroups are unable to engage because they lack opportunity [35], this lack may deepen their sense of social exclusion. Extant research indicates that institutional characteristics make volunteering more feasible for low-income minorities [37], but findings are not yet conclusive enough to provide a solid foundation for policy and practice interventions. Moreover, reasons for volunteering and the benefits it provides to vulnerable elders are not substantively different from those who are less economically vulnerable [38]. With empirical longitudinal evidence on the effects of social disengagement on depressive symptoms among the poor, our study adds weight to calls for developing organizational or environmental support systems, or both.

The current study provides several important implications for future research. The first implication affects the analytical approach employed to model group trajectories of depressive symptoms. In the current study, based on the strong existing evidence for gender-specific depression in old age, we modeled a conditional LCGA to identify a gender-contingent heterogeneous developmental pattern of depressive symptoms. In the current study, we aimed to explore the associations between health/social factors and trajectories of depressive symptoms, in the absence of established evidence related to the specific dynamic relations. This study’s findings provide initial evidence for associations between health/social engagement and whether trajectory groups of depressive symptoms vary by poverty status. Future research in this line should seek to determine whether health and social engagement were included as determining factors in different patterns of depressive symptom trajectories and how the dynamic varies by poverty status.

Additionally, this study provides meaningful direction for future cross-national research. Among less-developed countries, the empirical evidence regarding poverty and later-year depression is informing efficient strategies to address later-year depressive symptoms, particularly since, in many, poverty eradication is a national goal [1]. Previous studies found disparate groups of depressive symptom changes in developed countries, and in this study, we found similar heterogeneity in Korea. Since no cross-national comparative research yet exists, our findings need to be interpreted within the unique Korean context, which is often characterized as a mismatch between drastic cultural change caused by social isolation (i.e., a reduced role of filial support for aging parents) and a yet-to-be-stabilized old age pension system. This singular context produces an inverse relationship between poverty and later-year depressive symptoms.

In Korea, poor older adults have been identified as the most vulnerable subgroup, which our findings confirm dynamic patterns among health, social engagement, and depression are manifestations of this. Future empirical inquiries should explore whether the significance of social engagement we observed applies in less and more developed countries with different cultural and social-support contexts. For example, would a different society find different sub-populations among older adults vulnerable to depression (e.g., minorities, urban/rural)?

Another promising venue for future research is a detailed examination of disparate types of social engagement. In this exploratory study, we used a single indicator; however, mounting evidence suggests that the direction and strength of social engagement effects on depressive symptoms vary by type of social activity [39]. Future research should examine the extent to which different forms of participation affect depressive symptoms and whether the association varies across poverty groups. We acknowledge this study’s limitations. First, data does not contain information on death; thus, we could not control for attrition bias due to death. Death in old age is not a random event; rather, those who died may have had worsening health conditions. Ideally, a sensitive analysis should investigate a possibly differential association between health, social engagement, and death. It is a possibility that the association between declining health and trajectories of depressive symptoms may have been underestimated.

Second, data exclude older individuals residing in long-term care facilities, which introduces selection bias risk, given higher rates of depression among older individuals in hospitals or long-term care facilities [40]. Third, older individuals with health conditions may have limited ability to engage in social participation, leading to depressive symptoms. Identifying such complex associations between multiple health and social factors is beyond this study’s scope. Finally, in our current LCGA model, the variance and covariance estimates for the growth factors within each class were assumed to be fixed to zero. In preliminary analyses, after confirming that there were, indeed, several group trajectories, we moved on to a more advanced model, GMM, which allows variations in each subgroup. Unfortunately, the model did not converge. As allowing across-class variation in covariance matrices often creates convergence problems, improper solutions, and overall model instability, it is not unusual to decide to estimate only the intercept and not the slope. Although LCGA was the best viable analytical approach for the current study, findings of this study may not be generalizable to other studies.

## 5. Conclusions

Findings identifying differential patterns of later-year health and social risk and protective factors among poor and non-poor adults are an important contribution to geriatric literature, advancing knowledge regarding later-year depressive symptoms and laying the foundation for an efficient, targeted policy approach to ameliorating the persistent poverty-depression link.

## Figures and Tables

**Table 1:** Characteristics of depressive symptoms group trajectories among the poor (mean (standard deviation), or %) (*N* = 2461).

	Entire	High-to Moderate 8.61% (*n* = 212)	Slightly Increasing 44.13% (*n* = 1086)	Stable Low 36.77% (*n* = 905)	Stable High 6.50% (*n* = 160)	Steeply Increasing 3.98% (*n* = 98)	Statistics
**Demographics**							
Age	73.18	75.0 (7.1)	73.4 (6.3)	72.1 (5.9)	74.7 (6.0)	74.3(5.8)	F(4,2456) = 14.408 ***
Education (≤six years)	76.8%	81.1%	83.7%	64.4%	93.8%	76.5%	$x^{2}$(4) = 134.742 ***
Income status ($, log mean value)	4.0 (2.3)	4.0 (2.2)	4.0 (2.2)	4.1 (2.3)	4.1 (2.2)	3.3 (2.5)	F(4,2456) = 2.545 *
Constantly poor	62.0%	73.3%	61.3%	57.4%	75.8%	67.3%	$x^{2}$(4) = 28.481 ***
Marital status (ref. constantly married)	57.5%	49.5%	49.4%	74.5%	30.6%	51.0%	$x^{2}$(8) = 202.759 ***
Constantly unmarried	35.3%	45.3%	42.5%	19.7%	60.0%	36.7%	
Status changed	7.3%	5.2%	8.1%	5.9%	9%	12.2%	
**Health factors**							
Chronic diseases (ref. constantly no diseases)	18.1%	10.8%	15.7%	26.1%	5.0%	8.2%	$x^{2}$(8) = 107.160 ***
Constantly having diseases	70.0%	80.7%	72.9%	60.0%	90.6%	72.4%	
Increased	11.9%	8.5%	11.4%	13.8%	4.4%	19.4%	
Functional limitation (ref. constantly no disability)	61.8%	47.2%	62.9%	72.3%	40.2%	21.4%	$x^{2}$(12) = 218.328 ***
Constantly having disability	5.9%	9.7%	6.0%	3.7%	13.6%	7.1%	
Increase	20.4%	24.4%	19.6%	13.3%	31.1%	65.3%	
Decrease	11.8%	18.8%	11.6%	10.7%	15.2%	6.1%	
Cognition (ref. constantly normal)	26.3%	11.4%	18.8%	44.4%	6.1%	6.1%	$x^{2}$(12) = 332.610 ***
Constantly low and dementia risk	42.5%	60.2%	47.0%	24.9%	79.5%	61.2%	
Improved	10.8%	13.1%	10.8%	12.8%	3.0%	2.0%	
Declined	20.3%	15.3%	23.4%	17.9%	11.4%	30.6%	
**Social Engagement**							
Social participation (ref. constantly participated)	38.9%	18.8%	38.0%	49.6%	26.5%	14.3%	$x^{2}$(12) = 151.651 ***
Constantly non-participated	25.3%	40.3%	25.5%	18.6%	38.6%	32.7%	
Increased	17.1%	23.3%	18.4%	15.8%	12.9%	9.2%	
Decreased	18.7%	17.6%	18.1%	16.0%	22.0%	43.9%	
Contact frequency with close social network (ref. no change)	62.1%	55.1%	64.2%	63.3%	59.8%	48.0%	$x^{2}$(8) = 47.502 ***
Increased	17.7%	21.6%	16.2%	20.1%	15.2%	9.2%	
Decreased	20.2%	23.3%	19.7%	16.7%	25.0%	42.9%	

Note: Significance level of *p*-value * *p* < 0.05, ** *p* < 0.01, *** *p* < 0.001.

**Table 2:** Characteristics of depressive symptoms group trajectories in the non-poor group (mean (standard deviation), or %) (*N* = 1668).

	Entire	High to Moderate 5.3% (*n* = 88)	Steeply Increasing 11.5% (*n* = 192)	Stable Low 83.2% (*n* = 1388)	Statistics
**Demographics**					
Age	72.7 (6.3)	75.1 (7.3)	74.7 (6.7)	72.2 (6.1)	F(2,1665) = 20.247 ***
Education (≤six years)	66.7%	87.5%	79.7%	63.5%	$x^{2}$(2) = 37.924 ***
Income status ($, log mean value)	7.2 (0.5)	7.1 (0.5)	7.2 (0.6)	7.2 (0.5)	F(2,1665) = 2.279
Constantly non-poor	73.2%	60.3%	71.9%	74.1%	$x^{2}$(2) = 5.513
Marital status (ref. constantly married)	54.9%	29.5%	39.1%	58.6%	$x^{2}$(4) = 54.530 ***
Constantly unmarried	40.0%	67.0%	53.6%	36.4%	
Status changed	5.2%	3.4%	7.3%	5.0%	
**Health factors**					
Chronic diseases (ref. constantly no diseases)	19.5%	13.6%	6.3%	21.7%	$x^{2}$(4) = 35.964 ***
Constantly having diseases	67.1%	81.8%	78.6%	64.6%	
Status changed	13.4%	4.5%	15.1%	13.7%	
Functional limitations (ref. constantly no disability)	64.5%	46.6%	37.0%	69.9%	$x^{2}$(6) = 125.181 ***
Constantly having disability	5.8%	17.2%	8.3%	4.8%	
Increased	18.5%	13.8%	42.7%	14.8%	
Decreased	11.1%	22.4%	12.0%	10.4%	
Cognition (ref. constantly normal)	36.3%	10.3%	11.5%	41.6%	$x^{2}$(6) = 121.166 ***
Constantly low and dementia risk	33.1%	58.6%	57.3%	28.0%	
Improved	10.1%	17.2%	4.7%	10.7%	
Declined	20.4%	13.8%	26.6%	19.7%	
**Social factors**					
Social participation (ref. constantly participated)	49.9%	44.8%	33.9%	52.7%	$x^{2}$(6) = 55.933 ***
Constantly non-participated	18.5%	31.0%	31.8%	15.8%	
Increased	13.6%	8.6%	7.8%	14.7%	
Decreased	18.0%	15.5%	26.6%	16.8%	
Contact frequency with close social network (ref. no change)	60.1%	58.6%	50.5%	61.8%	$x^{2}$(4) = 26.013 ***
Increased	19.6%	19.0%	15.6%	20.3%	
Decreased	20.3%	22.4%	33.9%	18.0%	

Note: Significance level of *p*-value *** *p* < 0.001.

**Table 3:** Trajectories groups of depressive symptoms among the poor.

	High to Moderate		Slightly Increasing		Stable High		Steeply Increasing	
	Coefficient	Relative Risk Ratio	Coefficient	Relative Risk Ratio	Coefficient	Relative Risk Ratio	Coefficient	Relative Risk Ratio
**Demographics**								
Age	−0.031	0.969	−0.015	0.985	−0.062 *	0.940	−0.060 **	0.942
Education (≥7 years)	−0.051	0.951	−0.524 ***	0.592	−0.727	0.483	−0.064	0.938
Income status (Wave 1)	−0.006	0.994	−0.020	0.980	−0.008	0.992	−0.173 **	0.841
Exit from poverty	0.071	1.073	−0.057	0.945	−0.432	0.649	−0.379	0.684
Marital status (ref. constantly married)								
Constantly unmarried	0.270	1.310	0.685 ***	1.983	1.134 **	3.108	0.791 **	2.205
Status changed	0.768	2.154	0.634**	1.885	1.968 **	7.155	1.040 *	2.831
**Health factors**								
Chronic diseases (ref. constantly no diseases)								
Constantly having diseases	0.887	2.428	0.599 ***	1.821	2.052 **	7.787	0.952 *	2.591
Status changed	−0.269	0.765	0.334	1.397	0.511	1.666	1.160 *	3.189
Functional disability (ref. constantly no disability)								
Constantly having disability	−0.046	0.955	0.163	1.177	0.602	1.825	1.465 **	4.328
Increase	0.468	1.596	0.153	1.166	0.863 *	2.371	2.241 ***	9.404
Decrease	−0.253	0.776	−0.111	0.895	−0.392	0.676	0.390	1.476
Cognition (ref. constantly normal)								
Constantly low and dementia risk	1.409 *	4.093	−0.958 ***	2.606	1.964 **	7.129	1.831 ***	6.242
Improved	1.361 *	3.900	0.313	1.367	0.164	1.179	−0.362	0.696
Declined	0.937	2.551	0.921 ***	2.511	0.915	2.498	1.615 **	5.027
**Social factors**								
Social participation (ref. constantly participated)								
Constantly non-participated	−0.084	0.919	0.135	1.145	−0.322	0.724	0.992 **	2.696
Increased	−0.035	0.965	0.311	1.365	−0.771	0.462	0.771	2.161
Decreased	−0.214	0.807	0.009	1.009	−0.405	0.667	1.389 ***	4.011
Contact frequency with close social network (ref. no change)								
Increased	−0.323	0.724	−0.253	0.777	−0.564	0.569	−0.264	0.768
Decreased	−0.158	0.854	0.163	1.177	−0.119	0.888	0.565 *	1.759
W1 depression	1.014 ***	2.757	0.127 ***	1.135	0.891 ***	2.438	0.034	1.034

Note: Significance level of *p*-value * *p* < 0.05, ** *p* < 0.01, *** *p* < 0.001; Reference group: Stable low.

**Table 4:** Trajectory groups of depressive symptoms among the non-poor.

	High-to-Moderate		Steeply Increasing	
	Coefficient	Odd Ratio	Coefficient	Odd Ratio
Demographics				
Age	0.003	1.003	−0.005	0.995
Education (≥7 years)	−1.574	0.207	−0.179	0.836
Income status (W1)	−1.166 *	0.312	0.121	1.129
Exit from the poverty	−0.663	0.515	0.147	1.159
Marital status (ref. constantly married)				
Constantly unmarried	−0.040	0.961	0.356	1.428
Status changed	0.851	2.343	0.303	1.354
Health factors				
Chronic diseases (ref. constantly no diseases)				
Constantly having diseases	−1.045	0.352	0.886 **	2.425
Status changed	−0.566	0.568	0.647	1.910
Functional disability (ref. constantly no disability)				
Constantly having disability	0.130	1.139	0.447	1.563
Increase	−1.038	0.354	1.125 ***	3.080
Decrease	0.452	1.571	0.417	1.517
Cognition (ref. constantly normal)				
Constantly low and dementia risk	1.090	2.974	1.191 ***	3.289
Improved	0.383	1.467	0.230	1.259
Declined	0.748	2.113	1.308 ***	3.697
Social factors				
Social participation (ref. constantly participated)				
Constantly non-participated	−0.796	0.451	0.260	1.297
Increased	−0.492	0.611	−0.457	0.633
Decreased	0.057	1.059	0.186	1.204
Contact frequency with close social network (ref. no change)				
Increased	−0.474	0.623	−0.079	0.924
Decreased	−0.625	0.535	0.441 *	1.555
W1 depression	1.007 ***	2.736	0.116 ***	1.123

Note: Significance level of *p*-value * *p* < 0.05, ** *p* < 0.01, *** *p* < 0.001; Reference group: class 3.
